# Evaluation of Oral Toxicity of Palatinose/Fructooligosaccharide and Palatinose/Invert Sugar in Sprague–Dawley Rats

**DOI:** 10.3390/cimb48080827

**Published:** 2026-08-14

**Authors:** Seung-U Son, Hye-Ryung Park, Sue Jung Lee, Kwang-Soon Shin

**Affiliations:** 1 Precision Nutrition Research Group, Korea Food Research Institute, Wanju-gun 55365, Republic of Korea; suson@kfri.re.kr; 2 Department of Food Science and Biotechnology, Kyonggi University, 154-42 Gwanggyosan-ro, Suwon 16227, Republic of Korea; hrpark8512@gmail.com; 3 Natural Product Informatics Research Center, Korea Institute of Science and Technology, 679 Saimdang-ro, Gangneung 25451, Republic of Korea; suejung1209@kist.re.kr

**Keywords:** palatinose/invert sugar, palatinose/fructooligosaccharide, Sprague–Dawley rat, oral administration, toxicity

## Abstract

An evaluation of the safety profile of palatinose/fructooligosaccharide (P/FOS) and palatinose/invert sugar (P/IS), as novel low-glycemic and functional sweetener combinations, is essential for their application. This study aimed to evaluate the safety of 1000 mg/kg/day of P/FOS and 1000 mg/kg/day of P/IS through a 14-day repeated-dose oral toxicity study using male and female Sprague–Dawley (SD) rats. Clinical signs, such as body weight and organ weights, food/water consumption, hematology results, blood biochemical analysis results, urinalysis results, and histological changes in the liver and kidneys, were recorded in all SD rats. Barely any significant changes in food/water consumption, body weight, and organ weight were observed during the experimental period. Although there were some alterations in the results of hematological analysis, serum biochemical analysis, and urinalysis, these changes were not considered toxicologically significant within the normal ranges in both male and female SD rats administered with P/FOS and P/IS at 1000 mg/kg/day. Additionally, a histopathological analysis of the liver and kidneys showed no toxicological changes in P/FOS- and P/IS-treated SD rats of both sexes at the tested dose. Collectively, the above results confirmed that no overt treatment-related adverse effects of P/FOS and P/IS were observed under the tested conditions.

## 1. Introduction

Recently, influenced by changes in dietary habits, increasing life expectancy from medical advances, and modern lifestyle shifts, the incidence of metabolic diseases including obesity, diabetes, hypertension, and hyperlipidemia has continuously grown [1,2,3]. Carbohydrates, including sugars, are an essential source of energy for physiological and physical activities while also playing an important role in imparting sweetness and texture to foods [4,5]. Studies have shown that the excessive intake of sugar increases the incidence of chronic metabolic diseases, such as obesity, type 2 diabetes, hypertension, and cardiovascular diseases. The World Health Organization recommends that sugars should account for less than 10% of the total energy intake of the diet and suggests that a further reduction to below 5% can provide additional health benefits [6]. Accordingly, consumer interest has shifted toward sugar substitutes such as tagatose, stevia, allulose, and palatinose [7,8]. Palatinose is a disaccharide linked by an α-1,6-glycosidic bond between glucose and fructose, whereas sucrose is linked by an α-1,2-glycosidic bond between glucose and fructose [9,10].

Palatinose is produced via bioconversion through the enzymatic rearrangement of the glycosidic linkage from α-(1→2)-fructopyranoside to α-(1→6)-fructopyranoside [11]. The taste and appearance of palatinose are similar to those of sucrose, whereas its sweetness is about half that of sucrose. Moreover, the absorption rate of palatinose is slower than that of sucrose, being absorbed at one-fifth of the rate. Other properties of palatinose include a lower melting point (123–124 °C) than sucrose (160–185 °C), higher stability under acidic conditions, non-cariogenicity, and low solubility, and it is hardly fermented by environmental or oral microbes [10,12]. In a previous study, we demonstrated that the oral administration of palatinose significantly improves hyperglycemia through hepatic lipogenesis and cholesterol homeostasis compared to the oral administration of sucrose in C57BL/6J mice [13,14]. However, palatinose syrup possesses a lower solubility than sucrose [15], presenting disadvantageous crystallization issues during low-temperature and long-term storage [15]. This crystallization of palatinose raises concerns regarding the product quality assurance and stability for consumers.

On the other hand, fructooligosaccharides (FOSs), in which fructose units are homogenously linked via β-(2→1) glycosidic linkages, are non-digestible carbohydrates known to promote beneficial intestinal microbiota [16,17,18]. FOS consists of a mixture of several repeating sugars such as 1-ketose, nystose, and frutofuranosylnystose and possesses high solubility [19]. Additionally, invert sugar (IS) obtained from sucrose through acidic or enzymatic hydrolysis is one of the most commonly used sweeteners and is known to have a lower susceptibility to crystallization [20,21]. Based on the above background, the mixture of FOS or IS with palatinose was expected to compensate for the disadvantages of palatinose such as crystallization. However, a toxicity evaluation of palatinose/FOS (P/FOS) and palatinose/IS (P/IS) mixtures should be conducted to ensure food safety and provide toxicological data. In addition, to establish a sufficient safety margin for prospective human consumption, evaluating a dose limit of 1000 mg/kg/day, as outlined in OECD Guideline 407 for repeated-dose oral toxicity, is recommended for novel functional food ingredients. Therefore, in this study, we evaluated the safety of P/FOS and P/IS by assessing their toxicity in SD rat models. Overall, these findings are expected to serve as foundational safety data for the practical application and formulation of P/FOS and P/IS in the food industry.

## 2. Materials and Methods

### 2.1. Materials

**Figure 1:**
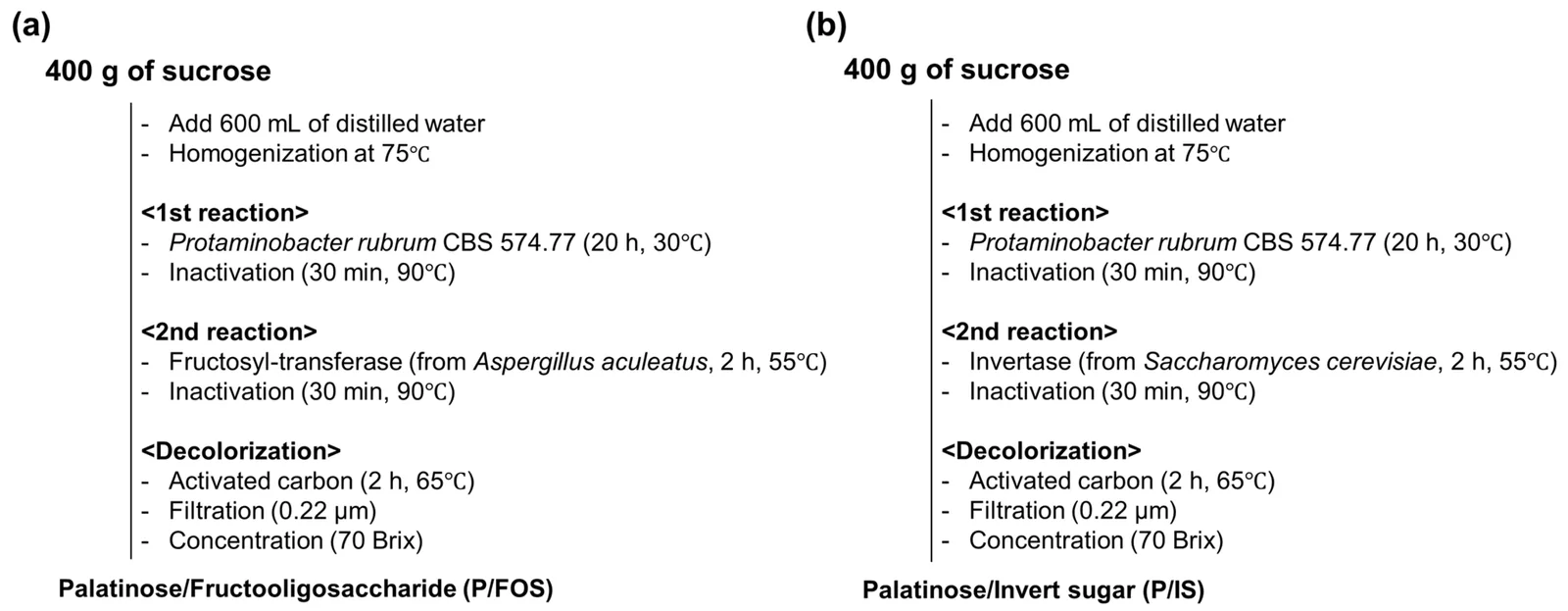
Manufacturing process of P/FOS (**a**) and P/IS (**b**). P/FOS, palatinose/fructooligosaccharide; P/IS, palatinose/invert sugar.

The P/FOS and P/IS samples used in this study were obtained from Neo Cremar Co. Ltd. (Seoul, Republic of Korea). Briefly, the 400 g of sucrose powder was dissolved in a 600 mL of distilled water, and homogenized using magnetic stirrer. Subsequently, for the 1st reaction, *Protaminobacter rubrum* CBS 574.77 was supplied to the sucrose solution for 20 h at 30 °C, and reactants was inactivated for 30 min at 90 °C. In the 2nd reaction, fructosyl-transferase and invertase (*Saccharomyces cerevisiae*) were used for P/FOS and P/IS, respectively (2 h, 55 °C). After that, decolorization was conducted using activated carbon for 2 h at 65 °C. The resulting solution was filtered using a 0.22 μm micro-filter and concentrated to 70 Brix using a vacuum rotary evaporator (EYELA, Tokyo, Japan). Consequently, the final products were named P/FOS (Figure 1a) and P/IS (Figure 1b). Additionally, the component sugars of P/FOS and P/IS were analyzed (Table S1 and Figure S1) based on the analytical conditions presented in Table S2, confirming purities of 83.8% and 92.8% on a dry matter basis, respectively.

### 2.2. Experimental Animals

Six-week-old male and female Sprague–Dawley (SD) rats were purchased from Saeron Bio (Uiwang, Republic of Korea) and provided with food and water ad libitum. All animals were housed under specific pathogen-free conditions/day and maintained at 23 ± 3 °C and 60 ± 10% humidity, with a cycle from 09:00 to 21:00 (12 h).

### 2.3. Experimental Design for Toxicity

After acclimatization for 7 days, the rats were weighed and randomly divided into three groups of 14 animals (7 males and 7 females) each: group 1 (control), group 2 (P/FOS), and group 3 (/IS). This group size was determined based on OECD guideline 407 recommendations to ensure sufficient statistical power while minimizing animal usage. Moreover, in accordance with the OECD guideline 407 for the limit test, the daily dosage was established at 1000 mg/kg/day. Rats in groups 2 and 3 were administered a single concentration of P/FOS and P/IS, respectively, at 1000 mg/kg (on a dry matter basis) via intragastric gavage once daily for 14 consecutive days. All pharmacological treatments were dissolved in distilled water. Rats in group 1 (control) were administered distilled water via intragastric gavage once daily throughout the study period. All animal experiments were performed in accordance with the guidelines of the Institutional Animal Care and Use Committee of Kyonggi University (Approval No. 2019-001, approved on 2 March 2019).

### 2.4. Clinical Signs of Toxicity, Body Weight, and Feed Consumption

Throughout the study period (14 days), all animals were observed individually once a day for clinical signs of toxicity and twice a day for mortality after oral administration. The body weight of all animals was measured once a day. Food and water consumptions were measured twice per week. The amounts of food and water were calculated before they were supplied to each cage, and their remnants were measured after 3 to 4 days to calculate the differences, which were regarded as average daily food and water consumption (g/rat/day).

### 2.5. Hematological Analysis

After fasting for 12 h, rats were euthanized via CO_2_ gas inhalation on the day of sacrifice, and blood was immediately collected from the abdominal aorta. For hematological analysis, 3 mL of blood was collected in an EDTA-2K-coated tube (Becton Dickinson, Franklin Lakes, NJ, USA). Hematological parameters, including white blood cell (WBC) count, red blood cell (RBC) count, hemoglobin (Hgb), hematocrit (HCT), mean corpuscular volume (MCV), mean corpuscular hemoglobin concentration (MCHC), platelet (PLT) count, red blood cell distribution width (RDW), and mean platelet volume (MPV), were determined using an automatic hematological analyzer (SYSMEX NX-1000, Sysmex Corporation, Kobe, Japan).

### 2.6. Serum Biochemical Analysis

For biochemical analysis, the obtained blood was centrifuged at 3000 rpm for 10 min to separate the serum. Serum biochemical analysis of the levels of glucose, albumin, alkaline phosphatase (ALP), blood urea nitrogen (BUN), direct bilirubin (D-BIL), total bilirubin (T-BIL), creatinine, triglyceride, total protein, alanine aminotransferase (ALT), and aspartate aminotransferase (AST) was performed using a Fuji Dri-Chem analyzer (3500, Fuji Photo Film Co., Osaka, Japan).

### 2.7. Urinalysis

On the day of the experiment, all rats from each group, which had been fasted overnight, were placed in metabolic cages for the collection of fresh urine. The collected urine samples were then analyzed for glucose, urinary urea nitrogen, T-BIL, and total protein levels using an automatic urine analyzer (HITACHI7180, Hitachi, Tokyo, Japan).

### 2.8. Histopathological Analysis

Tissues from the liver and kidney were collected and fixed in 4% formaldehyde solution for 24 h, routinely processed, and embedded in paraffin wax. Embedded tissues were sectioned at 3.5 μm using a microtome (Leica, Wetzlar, Germany) and stained with hematoxylin and eosin (H&E). All tissue sections were inspected using a microscope (Olympus BX53, Olympus Corp., Tokyo, Japan) at ×200, routine [22].

### 2.9. Statistical Analysis

All experimental results are presented as the mean ± standard deviation (SD). Significant differences among the groups were analyzed by one-way ANOVA followed by Tukey’s multiple comparisons test using GraphPad Prism 11.0 version (San Diego, CA, USA), with statistical significance defined at *p* < 0.05. An asterisk (*) indicates a significant difference between the control group and either the P/FOS or P/IS group, while a hash sign (#) represents a significant difference between the P/FOS and P/IS groups.

## 3. Results

### 3.1. Sugar Composition of P/FOS and P/IS

AnaAnalysis of the sugar compositions revealed distinct carbohydrate profiles for P/FOS and P/IS. In P/FOS, FOS was the major component at 31.2 ± 1.2%, followed by palatinose (19.2 ± 0.5%), glucose (17.0 ± 1.3%), sucrose (11.3 ± 0.5%), and fructose (5.1 ± 0.2%). In contrast, P/IS was predominantly composed of monosaccharides—fructose (38.6 ± 1.6%) and glucose (34.5 ± 1.3%)—along with palatinose (19.2 ± 0.7%) and a minor residual amount of sucrose (0.5 ± 0.3%), while FOS was not detected.

### 3.2. Survival and Clinical Signs of Toxicity

Throughout the experimental period (14 days), the control and P/FOS-treated, and P/IS-treated groups (1000 mg/kg/day) appeared healthy, and no mortality was observed in either male (*n* = 7) or female (*n* = 7) rats in any group. Moreover, in daily clinical observations following oral administration, no overt behavioral abnormalities or toxicity signs such as hypoactivity, hyperactivity, tremors, convulsions, or piloerection were observed in any treatment group.

### 3.3. Body Weight, Food Intake, and Water Consumption

**Figure 2:**
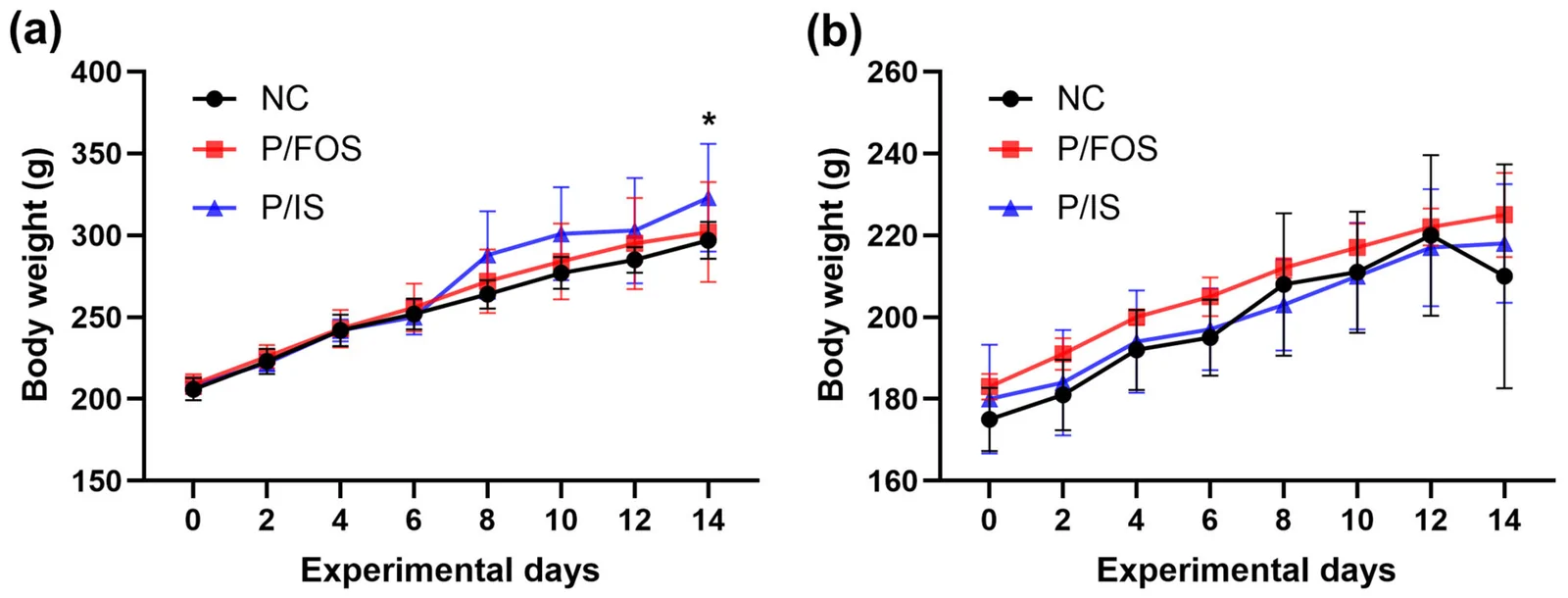
Body weights (g) in Sprague–Dawley (SD) rats administered orally with palatinose/fructooligosaccharide (P/FOS) and palatinose/invert sugar (P/IS) for the assessment of toxicity. Body weight of male (**a**) and female (**b**) SD rats. Data are expressed as mean ± SD (*n* = 7). Statistically significant differences compared to the control group are indicated by asterisks (* *p* < 0.05).

**Figure 3:**
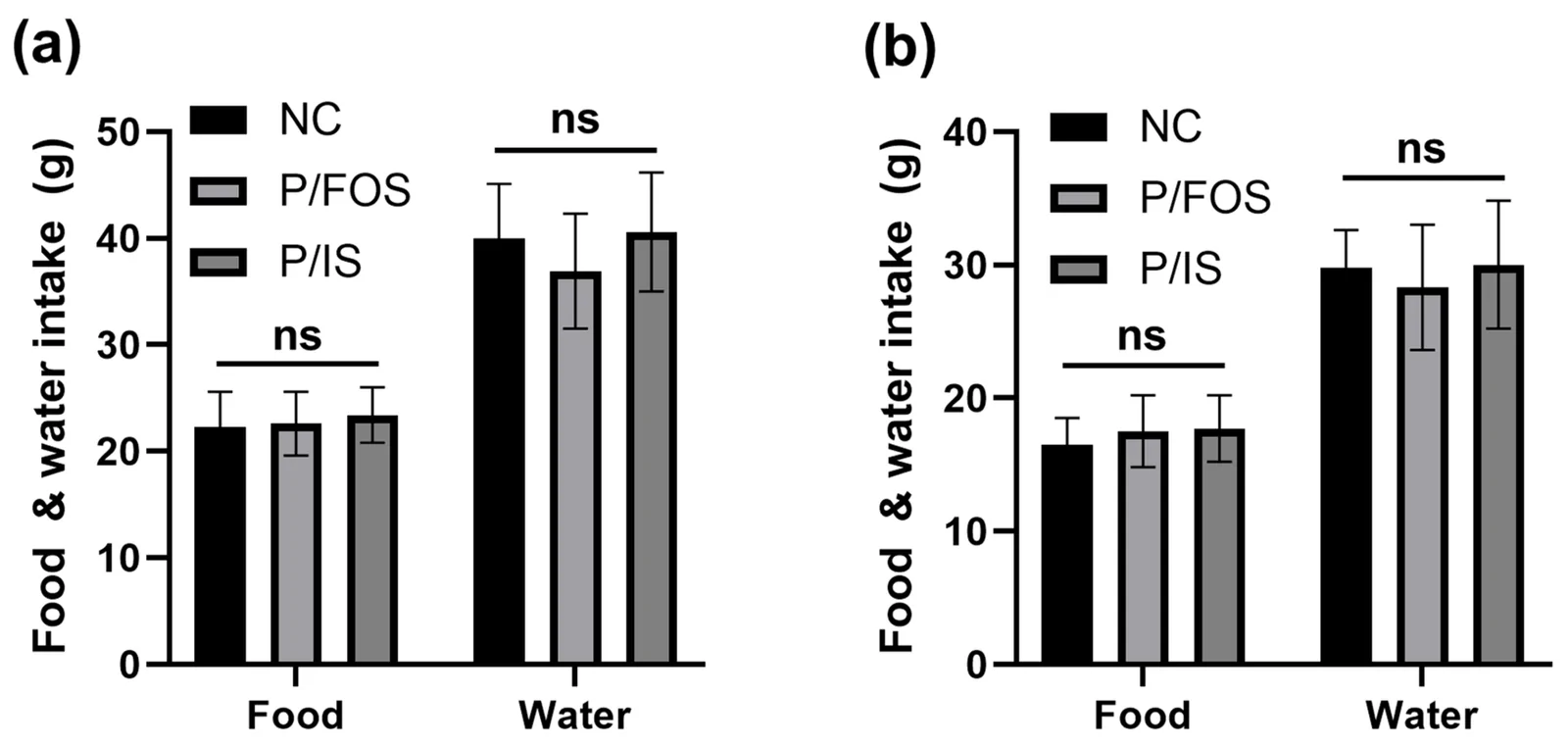
Food and water intake in Sprague–Dawley (SD) rats administered orally with palatinose/fructooligosaccharide (P/FOS) and palatinose/invert sugar (P/IS) for the assessment of toxicity. Body weight of male (**a**) and female (**b**) SD rats. Data are expressed as mean ± SD (*n* = 7). ns, not significant.

Throughout the experimental period (14 days), significant changes in body weight were barely observed among the control, P/FOS-treated, and P/IS-treated groups at a dose of 1000 mg/kg/day for both male and female rats, as shown in Figure 2. Furthermore, no significant differences in food intake and water consumption were noted among any of the groups for either sex (Figure 3).

### 3.4. Organ Weights

**Figure 4:**
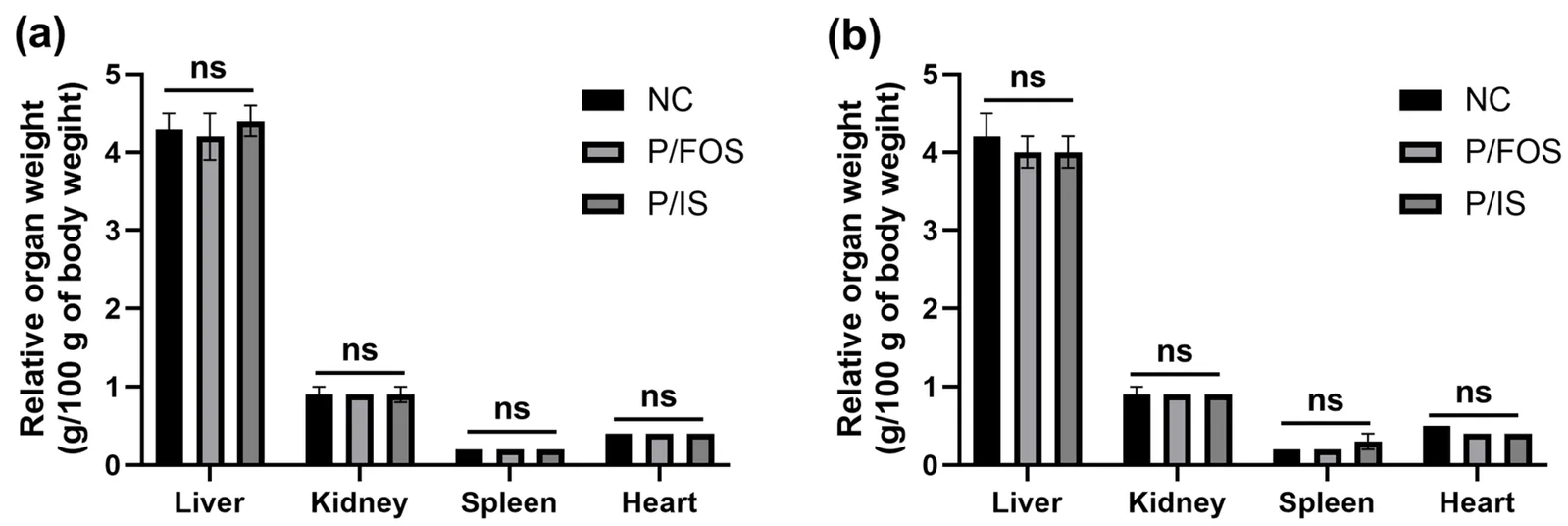
Relative organ weights in Sprague–Dawley (SD) rats administered orally with palatinose/fructooligosaccharide (P/FOS) and palatinose/invert sugar (P/IS) for the assessment of toxicity. Body weight of male (**a**) and female (**b**) SD rats. Data are expressed as mean ± SD (*n* = 7). ns, not significant.

After sacrifice, the liver, kidneys, spleen, and heart were excised to measure organ weights. There were no significant differences in the relative organ weights, including the liver, kidneys, spleen, and heart, in both male and female rats in the P/FOS- and P/IS-treated groups, compared to those of the control group (Figure 4).

### 3.5. Effects of P/FOS and P/IS on Hematological Parameters

Regarding hematological parameters (Table 1), no statistically significant differences were observed in any of the measured parameters, including WBC, RBC, Hgb, Hct, MCV, MCHC, PLT, RDW, and MPV, in male rats among all groups. Similarly, in female rats, most hematological parameters showed no remarkable alterations when compared to the control group. However, a statistically significant difference in MCHC levels was noted in female rats between the P/FOS-treated group (31.9 ± 0.4 g/dL) and the P/IS-treated group (31.3 ± 0.3 g/dL), although neither group differed significantly from the control group (31.5 ± 0.4 g/dL).

### 3.6. Effect of P/FOS and P/IS on Biochemical Parameters

Regarding the serum biochemical parameters (Table 2), several statistically significant alterations were observed in both male and female rats. In male rats, the P/IS-treated group showed significant decreases in the levels of BUN (15.8 ± 0.3 mg/dL), total protein (5.5 ± 0.1 g/dL), albumin (3.9 ± 0.1 g/dL), AST (63.3 ± 3.1 U/L), and ALT (27.4 ± 2.7 U/L) compared to the control group. The P/FOS-treated group also exhibited significant decreases in total protein (5.8 ± 0.2 g/dL) and AST (67.0 ± 3.6 U/L) levels compared to the control group. Furthermore, significant differences were observed between the two treated male groups, with the P/IS-treated group showing lower levels of BUN, total protein, albumin, and ALT than the P/FOS-treated group.

In female rats, the P/FOS-treated group showed a significant increase in glucose (124.4 ± 3.6 mg/dL) and albumin (4.8 ± 0.1 g/dL) levels compared to the control group. In contrast, the P/IS-treated group exhibited significant decreases in total protein (6.0 ± 0.1 g/dL) and albumin (4.2 ± 0.2 g/dL) levels compared to the control group. Interestingly, BUN levels were significantly decreased in both the P/FOS-treated (17.7 ± 0.8 mg/dL) and P/IS-treated (16.5 ± 0.5 mg/dL) groups compared to the control group (17.7 ± 0.4 mg/dL). Additionally, significant differences were noted between the female treatment groups, where glucose, total protein, and albumin levels in the P/IS-treated group were significantly lower than those in the P/FOS-treated group.

### 3.7. Effects of P/FOS and P/IS on Urinary Parameters

Regarding urinary parameters (Table 3), only male rats in the P/FOS-treated (2.4 ± 0.6 mg/dL) and P/IS-treated (2.6 ± 0.2 mg/dL) groups showed significant decreases in total protein levels compared to the control group (3.7 ± 0.7 mg/dL). In contrast, female rats in the P/FOS-treated and P/IS-treated groups did not exhibit remarkable differences in any urinary parameters, including glucose, urinary urea nitrogen, and total protein, when compared to the control group. Additionally, T-BIL was not detected in any of the experimental groups for either sex.

### 3.8. Effects of P/FOS and P/IS on Histopathological Parameters

**Figure 5:**
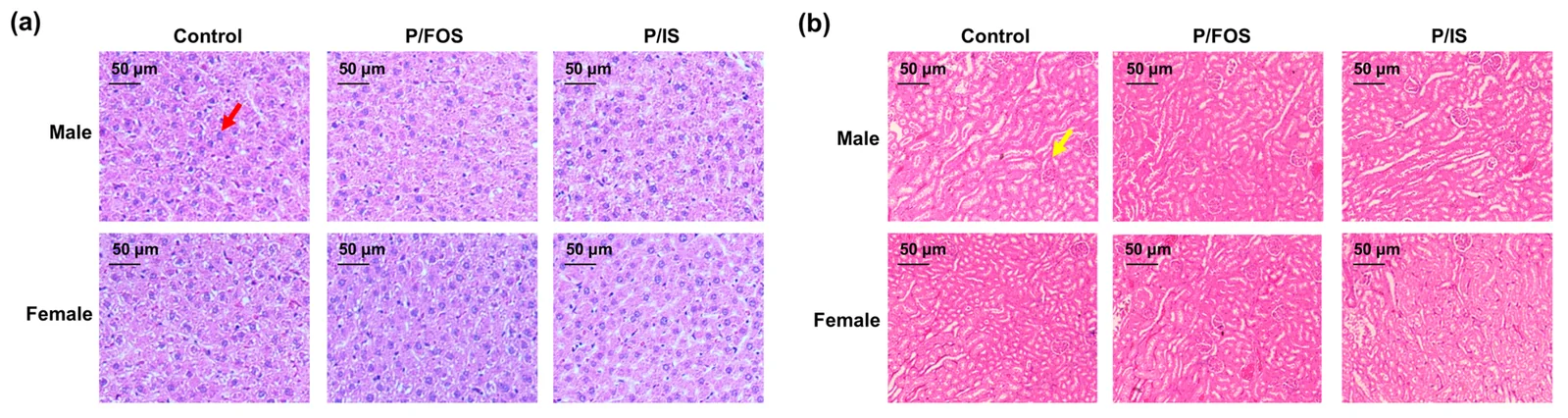
Histological examination of liver (**a**) and kidney (**b**) of male and female SD rats administered orally with P/FOS and P/IS for the assessment of toxicity. The red arrow indicates a hepatocyte, and the yellow arrow represents a glomerulus. Hematoxylin and eosin (H&E)-stained images of the liver and kidney of rats in the control, P/FOS-, and P/IS-treated groups. Original magnification, ×200. SD, Sprague–Dawley; P/FOS, palatinose/fructooligosaccharide; P/IS, palatinose/invert sugar.

Histopathological analysis revealed that the liver (Figure 5a) and kidneys (Figure 5b) of rats in the P/FOS- and P/IS-treated groups were similar to those observed in the control group rats. In the liver sections, the normal hepatic architecture and viable hepatocytes were well-preserved, with no evidence of edema, inflammatory cell infiltration, fibrosis or tissue degeneration. Similarly, the kidney sections demonstrated intact renal structures, including normal glomeruli, without any signs of cellular necrosis or edema. No pathological changes were observed in any organ of male or female rats exposed to P/FOS and P/IS at a dose of 1000 mg/kg/day.

## 4. Discussion

Palatinose is a representative sugar substitute for tagatose, allulose, and stevia. Palatinose is stable because it is produced not by chemical reactions but by enzymatic bioconversion from sucrose. The stability of the palatinose samples (P/FOS and P/IS) used in this study was established by adding FOS and IS to prevent the crystallization of palatinose. Interestingly, the crystallization of palatinose was completely inhibited by the addition of FOS or IS (Figure S2). Although both FOS and IS effectively prevent crystallization, they offer distinct functional advantages: FOS provides prebiotic benefits targeting gut health, whereas IS improves solubility and processing quality for general food applications. Therefore, evaluating their safety profile is an essential first step toward establishing their applicability. Standard OECD testing guidelines for chemicals, such as OECD Test Guideline 407 for repeated-dose oral toxicity, typically recommend a 28-day exposure period with multiple dose levels [23,24]. However, as an initial screening step prior to a formal 28-day evaluation, this preliminary study was designed using a 14-day repeated oral administration at a single limit concentration of 1000 mg/kg/day. This short-term design was aimed at establishing baseline safety and identifying any potential acute-to-subacute systemic toxicity, thereby laying the necessary toxicological foundation for future comprehensive long-term repeated-dose toxicity evaluations.

Changes in general behavior and body weight are critical parameters for evaluating the first signs of toxicity caused by drugs and chemicals [25]. In this toxicity analysis, rats administered a single concentration of 1000 mg/kg of P/FOS or P/IS showed no mortality, and no clinical signs such as changes in skin and eye color, general appearance, or relative organ weights were observed.

Since the liver is the main organ playing an important role in drug metabolism and biotransformation, liver function and tissue damage must be evaluated in a toxicity assessment. The serum biochemical parameters used to indicate liver tissue damage and liver function are glucose, albumin, ALP, BUN, D-BIL, T-BIL, creatinine, triglyceride, total protein, ALT, and AST. In this study, no significant difference was observed in the above-mentioned parameters between the P/FOS-administered group and the control group. Thus, we confirmed that P/FOS at a dose of 1000 mg/kg/day caused no hepatotoxicity in both male and female SD rats. In contrast, when evaluated using one-way ANOVA followed by Tukey’s post hoc test, the levels of total protein were significantly reduced in male SD rats in the P/IS-administered group at 1000 mg/kg/day compared to the control group.

To contextualize the blood biochemical variation in SD rats, published historical control data from 4-week toxicity studies—utilizing animals at a biological age (approx. 10 weeks old) comparable to those in our study (approx. 9 weeks old)—demonstrate that total protein levels exhibit a mean of 6.8 g/dL (range: 5.8–7.5 g/dL) for males and 6.8 g/dL (range: 5.7–7.8 g/dL) for females [26]. Relative to these historical baseline means, normal individual values naturally display physiological fluctuations ranging from −14.7% to +10.3% in males and −16.2% to +14.7% in females. In the present study, the administration of P/IS resulted in a 9.8% and 7.7% reduction in total protein levels compared to concurrent controls in males (5.5 ± 0.1 g/dL vs. 6.1 ± 0.1 g/dL, *p* < 0.0001) and females (6.0 ± 0.1 g/dL vs. 6.5 ± 0.1 g/dL, *p* < 0.0001), respectively, whereas P/FOS treatment induced a milder decrease (4.9% in males, *p* < 0.01; 1.5% in females, not significant). Although the decreases in both the P/FOS and P/IS groups reached statistical significance in males, and in the P/IS group in females, the magnitude of change remained well within the expected boundary of inherent biological variability for age-matched rats of this strain. Furthermore, this change was isolated and not accompanied by any corresponding alterations in other hepatic parameters or histopathological lesions. This was further supported by the H&E staining of the liver and kidneys, which clearly demonstrated that the administration of P/FOS and P/IS did not induce any pathological or toxicological alterations in either tissue.

In addition to total protein, a comprehensive blood biochemical analysis revealed several other statistically significant variations among the groups after multiple comparison tests. In male rats, the P/IS-treated group showed significant decreases in the levels of BUN, albumin, AST, and ALT compared to the control group, as well as lower levels of BUN, albumin, and ALT than the P/FOS-treated group. In female rats, the P/FOS-treated group exhibited a significant increase in glucose and albumin levels, whereas the P/IS-treated group showed a significant decrease in albumin compared to the control group. Furthermore, a significant reduction in BUN levels was captured in the P/IS-treated female group. From a toxicological perspective, tissue injury or systemic toxicity typically manifests as a pathological elevation of serum transaminases (AST and ALT) due to cellular leakage, or an increase in BUN indicating renal impairment. Therefore, the observed decreases in AST, ALT, and BUN in the treated groups do not indicate any adverse functional depression or structural damage. Although variations in glucose and albumin levels can reflect minor physiological acclimations or metabolic shifts, all of these altered parameters remained strictly within the historical physiological baseline ranges for SD rats of this age. Moreover, hematological and metabolic parameters often undergo transient physiological fluctuations in response to dietary carbohydrate shifts. Although minor statistical differences were captured in blood glucose levels, including a statistically significant increase in the female P/FOS group (*p* < 0.001), none of these parameters breached historical baseline boundaries or presented an adverse clinical point. Furthermore, because FOS functions as a non-digestible prebiotic fiber whereas IS supplies readily absorbable monosaccharides, the subtle metabolic variations between P/FOS and P/IS reflect a normal physiological acclimation to distinct sugar absorption kinetics rather than bone marrow toxicity or systemic metabolic disruption. Given the lack of any correlating clinical signs, functional impairment, or histopathological abnormalities, these statistically significant differences were determined to be toxicologically insignificant biological fluctuations. Furthermore, the non-adverse nature of these total protein fluctuations is further reinforced by the historical control data reported in another independent study [27], which confirms that the observed minor shifts do not represent toxicological impairment. Nevertheless, because this preliminary study focused primarily on general systemic toxicity following a 14-day repeated oral administration, specific gastrointestinal endpoints such as cecal characteristics, gut microbiota profiling, short-chain fatty acid production, and metabolomic analyses were not incorporated. Future long-term toxicity and efficacy studies should incorporate these gastrointestinal and microbiota-related parameters to fully elucidate the comprehensive physiological safety and gut-mediated metabolic impacts of P/FOS. Given that recent evidence highlights the tightly interconnected relationship between the gut microbiota and host metabolic pathways [28], integrating microbial and metabolomic profiles in future studies will be essential to provide deeper mechanistic insights into host–microbe interactions.

The hematopoietic system is one of the most sensitive targets for toxic substances and is an important index of physiological and pathological states [29]. Because blood is the major medium of transport for many nutrients and foreign bodies, components of blood, such as WBC, RBC, Hgb, HCT, MCV, MCHC, PLT, RDW, and MPV, are first exposed to toxic substances [30]. In the hematological analysis, no statistically significant differences were observed in any of the parameters evaluated following P/FOS or P/IS administration in either male or female SD rats. Therefore, these findings indicate no functional impairment of the hematopoietic system or bone marrow toxicity attributable to a P/FOS or P/IS administration of 1000 mg/kg/day.

In the results of urinalysis, the levels of total protein were significantly decreased in male SD rats in both the P/FOS-administered and P/IS-administered groups at 1000 mg/kg/day. In safety evaluations, a toxic response typically manifests as an abnormal increase in urinary protein excretion (proteinuria) resulting from renal filtration damage. Furthermore, total urinary protein levels in healthy rats are reported to be below 30 mg/dL [31,32]. Thus, the observed statistically significant decrease in total protein levels was considered toxicologically insignificant, especially given that the values were physiologically acceptable and well within the normal historical baseline.

The liver and kidneys are the organs most directly affected by toxic substances. Corroborating the biochemical and urinary findings, histopathological analysis showed no significant structural or toxicological changes in either the liver or kidneys. Specifically, in the liver sections, the normal hepatic architecture and viable hepatocytes were well-preserved, with no evidence of edema, inflammatory cell infiltration, fibrosis, or tissue degeneration. Similarly, the kidney sections demonstrated intact renal structures, including normal glomeruli and tubules, completely free from any signs of cellular necrosis or interstitial edema. However, histopathological evaluations in this study were limited to a qualitative assessment of representative tissue sections without formal blind scoring or lesion-severity grading. Furthermore, no significant changes in color, texture, and other abnormal signs were observed in male and female SD rats in the P/FOS- and P/IS-treated groups. From the above results, we concluded that neither P/FOS nor P/IS showed toxicity at 1000 mg/kg/day. Taken together, P/FOS and P/IS obtained through bioconversion did not induce significant toxicological changes, justifying the short-term safety profile of P/FOS and P/IS strictly under the limited experimental condition of a 14-day repeated oral administration at a single concentration of 1000 mg/kg/day in male and female SD rats. Rather than repeating the raw numerical values, the observed reductions in transaminases (AST and ALT) and total protein should be interpreted in a toxicological context. Hepatotoxicity typically induces hepatocellular leakage, leading to marked elevations in serum AST and ALT. The absence of enzyme elevation, combined with the intact hepatic microarchitecture in histopathology, confirms that P/FOS and P/IS do not exert hepatotoxic activity. Furthermore, previous studies on individual palatinose, FOS, and IS reported a high oral tolerance without structural tissue damage [10,33]. Our findings demonstrate that combining palatinose with FOS or IS does not produce a synergistic toxicity or altered metabolic burden.

Furthermore, a detailed comparative analysis between the P/FOS- and P/IS-treated groups revealed subtle statistical differences in specific hematological and serum biochemical parameters, including BUN, total protein, albumin, AST, ALT, and blood glucose. These observed variations can be directly correlated with the distinct sugar compositions as well as the physiological and metabolic characteristics of FOS and IS. FOS consists of non-digestible oligosaccharides that undergo gradual microbial fermentation in the lower gastrointestinal tract, whereas IS is composed of free glucose and fructose that are rapidly absorbed in the small intestine. Consequently, these structural and digestive differences naturally induce minor metabolic shifts in hepatic nutrient processing, protein synthesis, and systemic glucose regulation. Nonetheless, because all measured parameters in both the P/FOS and P/IS groups fluctuated strictly within normal historical physiological ranges without any accompanying histopathological injury, these variations represent benign physiological acclimations rather than toxicological disparities.

Despite these results, several limitations of the present study should be noted. First, this evaluation was performed utilizing a single concentration limit test at 1000 mg/kg/day, which precludes the characterization of dose-dependent toxicological responses or the determination of a formal no-observed-adverse-effect level (NOAEL). Second, because this investigation focused on a 14-day repeated oral administration period, it remains limited in predicting potential outcomes associated with extended or long-term systemic exposure. Third, organ weight measurements were restricted to the liver, kidneys, spleen, and heart, and histopathological examinations were performed exclusively on the liver and kidneys, without assessing other tissues (e.g., gastrointestinal tract, pancreas, reproductive organs, or lungs). Consequently, the overall safety conclusions regarding target-organ toxicity must be interpreted with respect to these examined organs. Fourth, because single-component comparator groups (palatinose-, FOS-, or IS-only) were not evaluated, this study cannot distinguish the individual physiological contributions of each component within the final mixtures. Therefore, the present findings reflect the overall preliminary safety of the specific P/FOS and P/IS formulations rather than palatinose alone. Nevertheless, establishing short-term oral safety at the limit dose provides essential clinical significance by confirming the absence of target-organ toxicity and verifying a safety margin for prospective human intake. Consequently, these preliminary findings serve as a necessary toxicological benchmark to justify and design future long-term subchronic/chronic exposure studies and clinical trials. Therefore, further systematic studies encompassing multiple dose gradients and prolonged exposure schedules are required to fully characterize the comprehensive long-term safety profiles and broader systemic interactions of P/FOS and P/IS.

## 5. Conclusions

To prevent the crystallization of palatinose, a toxicity test was conducted to evaluate the safety profiles of P/FOS and P/IS prepared by adding FOS and IS. Clinical signs of toxicity were assessed, and hematological, biochemical, and histopathological analyses were performed after the oral administration of P/FOS and P/IS for 14 days. Although biochemical parameters showed statistically significant changes in the treated groups, all values remained within normal physiological ranges without toxicological relevance. Furthermore, no significant abnormalities were observed in the histopathological analysis. Consequently, under the specific conditions of this 14-day study, the repeated oral administration of P/FOS and P/IS at 1000 mg/kg/day resulted in no overt treatment-related adverse effects in SD rats. These findings provide foundational baseline safety data to support further comprehensive long-term toxicity evaluations and potential applications of P/FOS and P/IS as novel alternative sweeteners.

## Figures and Tables

**Table 1:** Hematological parameters in Sprague–Dawley (SD) rats administered orally with palatinose/fructooligosaccharide (P/FOS) and palatinose/invert sugar (P/IS) for the assessment of toxicity.

Hematological Parameters	Groups for Assessment of Toxicity		
	Control	P/FOS	P/IS
Male			
WBC (k/μL)	15.4 ± 2.0	13.5 ± 2.2	13.9 ± 2.2
RBC (M/μL)	7.2 ± 0.5	7.5 ± 0.3	7.3 ± 0.3
Hgb (g/dL)	16.2 ± 0.7	16.4 ± 0.5	16.1 ± 0.6
Hct (%)	50.6 ± 2.1	50.9 ± 1.4	50.5 ± 1.7
MCV (fL)	60.2 ± 2.1	58.3 ± 0.7	59.5 ± 1.5
MCHC (g/dL)	32.0 ± 0.8	32.2 ± 0.5	31.9 ± 0.7
PLT (k/μL)	1038.4 ± 120.5	1031.9 ± 236.5	921.4 ± 171.9
RDW (%)	12.9 ± 0.8	13.2 ± 0.7	13.4 ± 0.9
MPV (fL)	9.6 ± 0.2	9.7 ± 0.4	10.0 ± 0.5
Female			
WBC (k/μL)	12.3 ± 1.5	11.4 ± 4.1	13.2 ± 2.2
RBC (M/μL)	6.9 ± 0.8	7.3 ± 0.4	7.3 ± 0.3
Hgb (g/dL)	14.8 ± 1.3	15.6 ± 0.9	15.4 ± 0.4
Hct (%)	47.0 ± 4.5	49.0 ± 0.9	49.3 ± 1.3
MCV (fL)	67.7 ± 1.6	67.2 ± 1.7	67.2 ± 1.0
MCHC (g/dL)	31.5 ± 0.4	31.9 ± 0.4	31.3 ± 0.3 ^#^
PLT (k/μL)	881.9 ± 181.8	951.0 ± 206.8	947.3 ± 203.5
RDW (%)	11.8 ± 0.7	12.5 ± 0.9	12.6 ± 0.5
MPV (fL)	9.8 ± 0.4	9.6 ± 0.7	10.0 ± 0.3

Data are expressed as mean ± SD (*n* = 7). A hash sign (#) indicates a significant difference between the P/FOS and P/IS groups (^#^ *p* < 0.01).

**Table 2:** Blood biochemical parameters in Sprague–Dawley (SD) rats administered orally with palatinose/fructooligosaccharide (P/FOS) and palatinose/invert sugar (P/IS) for the assessment of toxicity.

Blood Biochemical Parameters	Groups for Assessment of Toxicity		
	Control	P/FOS	P/IS
Male			
Glucose (mg/dL)	127.1 ± 2.7	127.0 ± 3.8	125.1 ± 2.8
ALP (U/L)	189.4 ± 42.7	187.7 ± 26.6	188.1 ± 39.9
BUN (mg/dL)	17.5 ± 0.9	16.7 ± 0.5	15.8 ± 0.3 ***^,#^
Creatinine (mg/dL)	0.2 ± 0.0	0.2 ± 0.0	0.2 ± 0.0
Total protein (g/dL)	6.1 ± 0.1	5.8 ± 0.2 **	5.5 ± 0.1 ****^,##^
Albumin (g/dL)	4.4 ± 0.2	4.4 ± 0.1	3.9 ± 0.1 ****^,####^
T-BIL (mg/dL)	0.3 ± 0.0	0.2 ± 0.0	0.2 ± 0.0
D-BIL (mg/dL)	0.1 ± 0.0	0.1 ± 0.0	0.1 ± 0.0
Triglyceride (mg/dL)	72.4 ± 7.8	79.8 ± 11.4	66.4 ± 18.3
AST (U/L)	72.7 ± 4.7	67.0 ± 3.6 *	63.3 ± 3.1 ***
ALT (U/L)	34.9 ± 2.7	36.0 ± 2.7	27.4 ± 2.7 ***^,####^
Female			
Glucose (mg/dL)	114.5 ± 4.0	124.4 ± 3.6 ***	114.2 ± 2.8 ^###^
ALP (U/L)	137.2 ± 11.5	123.2 ± 23.4	114.4 ± 34.1
BUN (mg/dL)	17.7 ± 0.4	17.7 ± 0.8	16.5 ± 0.5 **^,##^
Creatinine (mg/dL)	0.2 ± 0.0	0.2 ± 0.0	0.2 ± 0.0
Total protein (g/dL)	6.5 ± 0.1	6.4 ± 0.2	6.0 ± 0.1 ****^,###^
Albumin (g/dL)	4.5 ± 0.3	4.8 ± 0.1 *	4.2 ± 0.2 *^,###^
T-BIL (mg/dL)	0.3 ± 0.0	0.4 ± 0.1	0.3 ± 0.0
D-BIL (mg/dL)	0.1 ± 0.0	0.1 ± 0.0	0.1 ± 0.0
Triglyceride (mg/dL)	36.6 ± 16.0	56.2 ± 19.0	46.5 ± 11.7
AST (U/L)	61.8 ± 1.5	63.3 ± 3.0	61.4 ± 5.2
ALT (U/L)	27.6 ± 2.5	31.3 ± 2.9	27.9 ± 3.3

Data are expressed as mean ± SD (*n* = 7). Statistically significant differences compared to the control group are indicated by asterisks (* *p* < 0.05, ** *p* < 0.01, *** *p* < 0.001, and **** *p* < 0.0001). A hash sign (#) indicates a significant difference between the P/FOS and P/IS groups (^#^ *p* < 0.05, ^##^ *p* < 0.01, ^###^ *p* < 0.001, and ^####^ *p* < 0.0001).

**Table 3:** Urinalysis in Sprague–Dawley (SD) rats administered orally with palatinose/fructooligosaccharide (P/FOS) and palatinose/invert sugar (P/IS) for the assessment of toxicity.

Urinalysis	Groups for Assessment of Toxicity		
	Control	P/FOS	P/IS
Male			
Glucose (mg/dL)	15.7 ± 5.3	20.3 ± 3.9	16.7 ± 9.6
Urinary urea nitrogen (mg/dL)	2815.1 ± 503.8	2675.6 ± 180.6	2481.0 ± 322.8
Total protein (mg/dL)	3.7 ± 0.7	2.4 ± 0.6 ***	2.6 ± 0.2 **
T-BIL (mg/dL)	nd	nd	nd
Female			
Glucose (mg/dL)	14.9 ± 2.1	14.5 ± 7.6	15.4 ± 5.7
Urinary urea nitrogen (mg/dL)	2579.7 ± 674.0	2458.1 ± 799.6	2550.5 ± 1355.1
Total protein (mg/dL)	2.3 ± 1.1	2.1 ± 1.0	2.5 ± 0.7
T-BIL (mg/dL)	nd	nd	nd

Data are expressed as mean ± SD (*n* = 7). Statistically significant differences compared to the control group are indicated by asterisks (** *p* < 0.01, and *** *p* < 0.001). nd, not detected.

## Data Availability

The dataset is available upon request from the authors.

## Supplementary Materials

The following supporting information can be downloaded at https://www.mdpi.com/article/10.3390/cimb48080827/s1.

## Abbreviations

ALP	Alkaline phosphatase
ALT	Alanine aminotransferase
AST	Aspartate aminotransferase
BUN	Blood urea nitrogen
D-BIL	Direct bilirubin
FOS	Fructooligosaccharide
HCT	Hematocrit
Hgb	Hemoglobin
IS	Invert sugar
MCHC	Mean corpuscular hemoglobin concentration
MCV	Mean corpuscular volume
MPV	Mean platelet volume
P/FOS	Palatinose/FOS mixture
P/IS	Palatinose/IS mixture
PLT	Platelet count
RBC	Red blood cell count
RDW	Red blood cell distribution width
SD	Standard deviation
SD rat	Sprague–Dawley rat
T-BIL	Total bilirubin
TP	Total protein
WBC	White blood cell count
