# Supplementary material for: Evaluation of Oral Toxicity of Palatinose/Fructooligosaccharide and Palatinose/Invert Sugar in Sprague–Dawley Rats
Source: Curr Issues Mol Biol. 2026 Aug 14;48(8):827. doi: 10.3390/cimb48080827 (PMC13511687; doi:10.3390/cimb48080827)
Supplement: Supplementary file 1 [file cimb-48-00827-s001.zip › cimb-4465006-supplementary.pdf]

Table S1. Sugar composition of of P/FOS and P/IS

Sugar composition	Contents (%)	
	P/FOS	P/IS
Glucose	17.0 ± 1.3	34.5 ± 1.3
Fructose	5.1 ± 0.2	38.6 ± 1.6
Palatinose	19.2 ± 0.5	19.2 ± 0.7
Sucrose	11.3 ± 0.5	0.5 ± 0.3
Fructooligosaccharide	31.2 ± 1.2	-

Table S2. Analysis conditions on sugar composition of P/FOS and P/IS

	RID-HPLC	
Column	Develosil C30-UG-5	YMC-pack polyamine
Mobile phase	Water	64% acetonitrile
Flow rate	0.5 mL/min	1.0 mL/min
Column temperature	30℃	30℃
Detector temperature	45℃	45℃
Injection volume	20 μL	20 μL

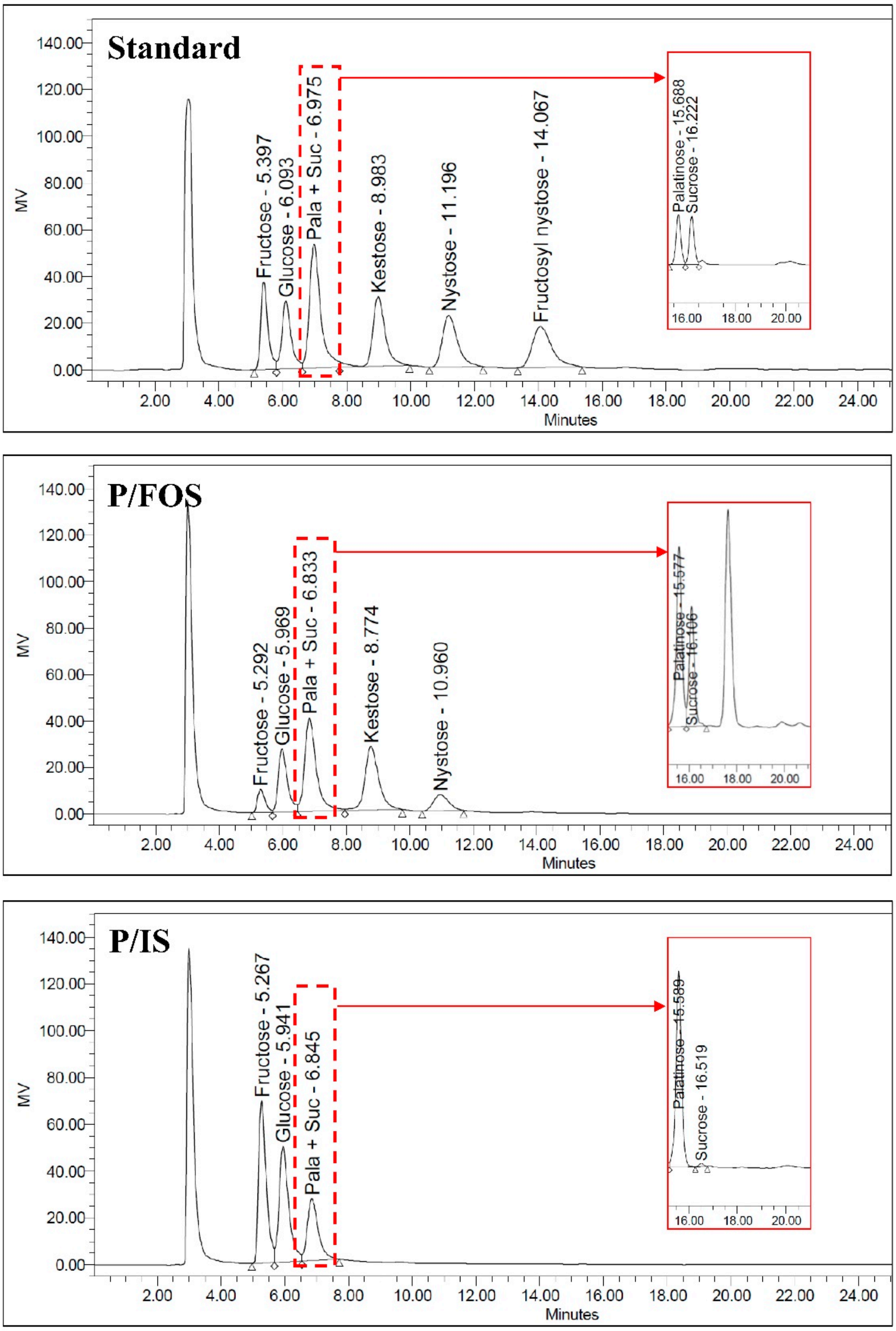

Figure S1. Sugar composition analysis of P/FOS and P/IS through refractive index detector-high performance liquid chromatography equipped with YMC-Pack Polyamine column (black frame) and Develosil C30-UG-5 column (red frame).

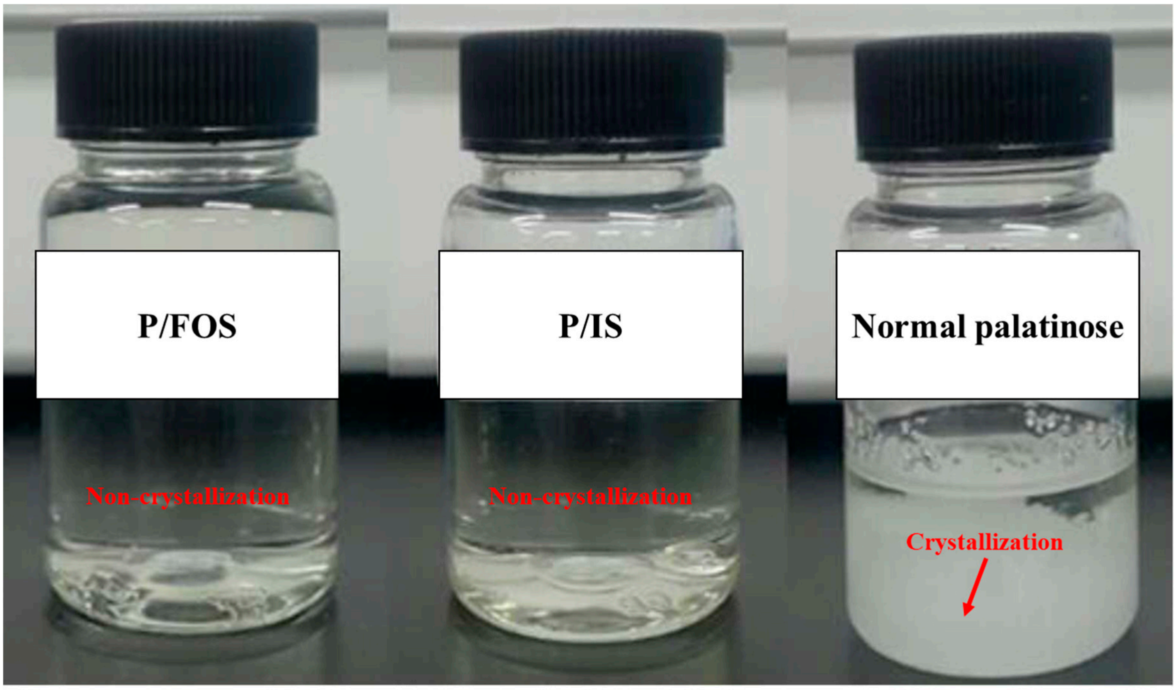

Figure S2. Effect of long-term (6 months, 25°C) storage on crystallization of P/FOS, P/IS, and normal palatinose
